# Supplementary material for: The value of lymphocyte to monocyte ratio in the prognosis of head and neck squamous cell carcinoma: a meta-analysis
Source: PeerJ. 2023 Sep 11;11:e16014. doi: 10.7717/peerj.16014 (PMC10501369; doi:10.7717/peerj.16014)
Supplement: Supplemental Information 3 [file peerj-11-16014-s003.docx]

The rationale for conducting the systematic review/meta-analysis: The association between different hematological markers is related to the prognosis of cancer, such as the rate of neutrophils to lymphocytes (NLR) and the rate of lymphocytes to monocytes (LMR) have been widely used in the prediction of various cancers. These hematological indicators are cheaper, easier to obtain, and more convenient for clinical application. Recently, it has been reported that LMR plays a crucial role in evaluating the prognosis of head and neck malignant tumors. However, the value of LMR in evaluating the prognosis of squamous cell carcinoma of head and neck (SCCHN) is not clear, and the conclusions of different reports are not completely consistent. Therefore, we conducted a meta-analysis to assess the value of the lymphocyte-to-monocyte ratio in predicting the prognosis of squamous cell carcinoma of head and neck. The contribution that it makes to knowledge considering previously published related reports, including other meta-analyses and systematic reviews: Based on findings from previous studies, LMR was associated with the prognosis of head and neck cancer. However, their studies lack high-quality prospective research. The function of the LMR in squamous cell carcinoma of head and neck is, however, unclear. This study provides a complete explanation of the outcomes. We found that lymphocytes and monocytes might forecast survival in SCCHN. Low LMR was correlated with poor prognosis in patients with SCCHN. The meta-analysis indicates that lymphocytes and monocytes might forecast survival in SCCHN. Low LMR was associated with poor prognosis in patients with SCCHN. As broadly accessible and economical markers, hematological parameters might accelerate prognosis forecast in human with SCCHN. It has a good prospect of clinical application.
